# Tracking Traction Force Changes of Single Cells on the Liquid Crystal Surface

**DOI:** 10.3390/bios5010013

**Published:** 2015-01-05

**Authors:** Chin Fhong Soon, Kian Sek Tee, Mansour Youseffi, Morgan C. T. Denyer

**Affiliations:** 1 Biosensor and Bioengineering Laboratory, MiNT-SRC, Universiti Tun Hussein Onn Malaysia, 83000 Batu Pahat, Malaysia; E-Mail: tee@uthm.edu.my; 2 School of Engineering, Design and Technology-Medical Engineering, University of Bradford, BD7 1DP Bradford, UK; E-Mail: m.youseffi@bradford.ac.uk; 3 School of Medical Sciences, University of Bradford, BD7 1DP Bradford, UK; E-Mail: m.denyer@bradford.ac.uk

**Keywords:** liquid crystals, cell traction force transducer, keratinocytes, cell force mapping, cell translocation

## Abstract

Cell migration is a key contributor to wound repair. This study presents findings indicating that the liquid crystal based cell traction force transducer (LCTFT) system can be used in conjunction with a bespoke cell traction force mapping (CTFM) software to monitor cell/surface traction forces from quiescent state in real time. In this study, time-lapse photo microscopy allowed cell induced deformations in liquid crystal coated substrates to be monitored and analyzed. The results indicated that the system could be used to monitor the generation of cell/surface forces in an initially quiescent cell, as it migrated over the culture substrate, via multiple points of contact between the cell and the surface. Future application of this system is the real-time assaying of the pharmacological effects of cytokines on the mechanics of cell migration.

## 1. Introduction

Re-epithelialization during wound repair is very dependant on epithelial cell migration into the wound site [1]. Understanding the process of wound repair has significant implications for treatment. One key aspect of the process is that of gaining an understanding of cell migration related cell/surface traction forces. A number of cell traction force systems have been developed mostly around the use of microscopy to monitor cell-induced wrinkles or marker displacement in soft polymer culture substrates [2,3,4,5,6]. Unfortunately the initial work by Harris *et al.*, 1980 [7] indicated that these systems could be used to monitor cell traction forces, but could not be used to accurately quantify the forces generated. The functionality of wrinkle based force measurement systems was subsequently enhanced by use of thinner and higher compliance silicon rubber [5]. Unfortunately, due to the cross-linking in these polymers, cell contractions induced non-linear chaotic wrinkles [5,8] making quantitative qualitative measurements complex rendering these systems suitable for only qualitative assessment of quiescent cell traction forces [9,10]. This problem is also exacerbated by polymers such as polydimethylsiloxane (PDMS) and silicon rubber [7]. PDMS behaves linear elastic at strains less than 25% and became non-linear at larger strains [11,12]. This is a factor needs to be considered in measuring cell traction forces based on PDMS micropillar because large deformations of PDMS micropillars are usually observed when cells adhered to the micropillars [13]. Low spatial resolution of the micropillars is a drawback of this technique in facilitating the cell to migrate [10]. In addition, traction force microscopy (TFM) is another technique used to quantify cell traction force based on displacement of randomly distributed fluorescence particles or markers in elastic polymer that were traced using image correlation approach [14]. However, the primary flaw of TFM is having low signal to noise ratio due to the randomly distributed markers and interdependence of cell induced displacement field due to the propagation of energy throughout the entire surface of the substrates [10,15].

The liquid crystal based cell traction force transducer (LCTFT) was first developed in 2012 by our group [16]. The liquid crystals in this system, function by providing a biocompatible, thermally stable (0–50 °C), substrate with a linear viscoelasticity at low shear rate (<1 s^−1^), that can support cell adhesion and growth [17,18]. The cells can be cultured on the liquid crystal surface without addition of extracellular matrix (ECM) proteins. Our previous study showed that keratinocytes could functionalize the liquid crystal (LC) surface using self-generated provisional ECM proteins [19]. In addition, this system allows the monitoring of reversible cell induced liquid crystal (LC) deformations. The spatial sensitivity of the system is such that it is capable of monitoring LC wrinkles arising from discrete focal contacts, where the length of wrinkles is directly related to the magnification of the generating forces (CTFs). That cells attach to a surface via multiple focal contacts means that the LCTFT system is sensitive enough to monitor forces generated from multiple sites enabling the generation of a cell traction force map. In this system, the relationship between LC deformation length and the CTFs is estimated by applying Poisson’s ratio of a deformation and Hooke’s theorem [16]. The LCTFT based system is very different to soft polymer based systems [15,20] because measurements rely on monitoring force induced organizational changes of mesogens, and is thus not constrained by the limitations associated with cross-linking. To date, the LC based cell traction force measurement system has enabled the investigation of cell surface forces between 10 to 120 nN in quiescent cells only [16]. For monitoring dynamic forces of live cells during migration, the sensitivity of this technique is yet to be determined. With this in mind, this study examines how the system can be combined with time-lapse photo-microscopy to enable the spatio-temporal resolution of cell surface traction forces in live human keratinocyte (HaCaT) type cells.

## 2. Experimental Section

### 2.1. Preparation of Liquid Crystal Substrate

The preparation of the cholesteryl ester liquid crystal mixtures and HaCaT cells were prepared as previously described in Soon *et al.* [18,19]. Cell suspensions at a cell density of 500 cells/cm^2^ were plated in Petri dishes containing liquid crystal (LC) coated cover slips and incubated at 37 °C for 24 h. After incubation, the cells cultured on the liquid crystals were found adhering and contracting, while inducing localized deformation lines in the LC surfaces.

### 2.2. Preparation of Human Keratinocyte Cell Lines

Human keratinocyte cell lines (HaCaT) were kindly provided by Dr. Steve Britland (University of Bradford, UK) and maintained in a 25 cm^2^ tissue culture (TC) grade cell culture flasks. On reaching confluence, cells were split as described previously [18] and re-suspended in 5 mL of RPMI-1640 media. Cells were either re-plated in a 25 cm^2^ TC grade culture flasks at a cell density of 1.5 × 10^4^ cells/cm^2^ or used for further experiments.

### 2.3. Culturing Cells on the Liquid Crystal Substrates

Cells were seeded onto a liquid crystal coated substrate placed in a Petri dish at a density of 500 cells/cm^2^. Following plating, the Petri dish was added with 6 mL of RPMI-1640 cell culture media and incubated at 37 °C for 24 h. After incubation, the responses of the LC to cell adhesion were studied in a GX-XDS2 phase contrast microscope at 25 × magnification (NA = 0.45) and photomicrographs were captured with a GT Vision CX digital camera linked to Scion Imaging Software. A cell with rounded morphology was selected and images of this cell were acquired every 5 min over a period of half an hour.

### 2.4. Staining for Actin Fibers and Vinculin Accumulations

After incubated on LC coated substrate at 37 °C for 24 h, the substrates was washed twice with Hanks Balanced Salt Solution (HBSS, Sigma Aldrich, Dorset, UK) fixed in 1% formaldehyde in HBSS for 6 min, rinsed a further two times in HBSS and incubated in 0.1% Triton X-100 for 3 min to permeabilized the membranes. F-actin staining was achieved by incubating the substrate in 1 µg/mL of Fluorescence Isothiocyanate (FITC) labeled Phalloidin solution (Sigma Aldrich) in HBSS for 45 min [16]. 

This final incubation was followed by another three washes in HBSS. As a contrast nuclear staining was undertaken via incubation in a solution of 4′6-diamidino-2-phenylindole-2HCl (DAPI) dihydrochloride at a concentration of 0.1 µg/mL in HBSS (Sigma Aldrich) for 15 min.

HaCaT cells cultured on LC coated substrates were also immuno-stained for vinculin expression using a procedure reported by Clubb *et al.* [21] and stained cells were imaged via a plan fluar lens of a Nikon Eclipse 80i fluorescence microscope (N.A of 1.3, 40× magnification) under dark field (DF) illumination. Photomicrographs were acquired using a digital camera and associated ACT-2u software. Blue (nuclei) and green (actin and vinculin) staining images were digitally merged using ImageJ software. All fluorescence staining experiments were repeated in triplicates.

### 2.5. Quantification of Cell Traction Forces Using Cell Traction Force Mapping Software

The custom-built cell traction force mapping software (CTFM) was developed in the MATLAB Integrated Development Environment (IDE). The software was applied to map localized cell traction forces in nano-newton (nN) based on the relationship of CTF-deformation established in Soon *et al.* [16]. The full details of the design and execution of the program can be found in the supplement of our previous publication [16]. The time resolved cell traction force responses graph was fitted using the line plot tool available in Microsoft Excel software.

## 3. Results and Discussion

**Figure 1:**
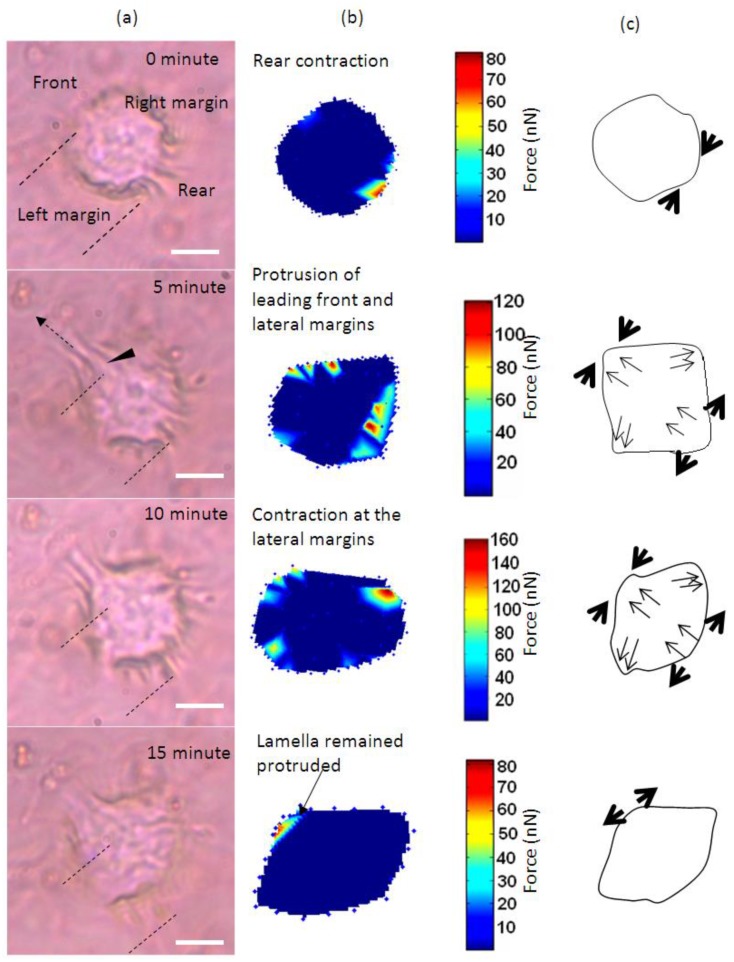
Time-base tractions of a keratinocyte on a liquid crystal based cell traction force transducer (LCTFT) displayed in (**a**) phase contrast micrographs which were taken at 0, 5, 10 and 15 min of monitoring. The broken line arrow indicates the direction of movement and the dotted lines are the position of reference for the cell; (**b**) The associated distribution of traction forces; (**c**) The directions of forces as shown with thick arrows and the thin arrows represent the direction of the forces and actin bundles flow, respectively. The scale bar in pseudo color represents the magnitude of forces in nano-newton. (Scale bar: 20 µm).

Figure 1 shows the time-lapse photo microscopy of a cell attached and migrated across the LC surface. As the cell migrate, cell traction forces translated into deformations that transiently rose and decayed over the LC surface. These deformations were quantified and rendered as a force distribution map according to the method reported in our previous work [16]. As shown in Figure 1a, cell area was divided into four regimes that are the trailing edge (rear), the leading edge (front) and the two lateral flanks (margins).

**Figure 2:**
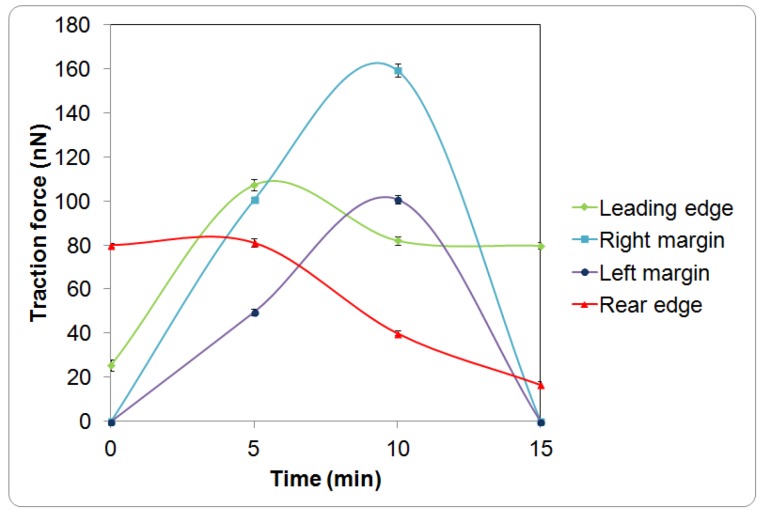
Time response curves of the traction force (mean ± standard deviation) measured at different regions of a single keratinocyte as presented in Figure 1.

The cell initially expressed rounded morphology (Figure 1a, 0 min) and demonstrated with moderate pinching forces at the rear of the cell (80 nN peak, Figure 1b,c, 0 min). At the same period of time, a small increase in force (peak 25 nN) was observed at the leading edge of the cell (Figure 1b and Figure 2). However, the lateral margins were in the quiescent state without extension of lamellipodia and no expression of traction forces was detected (Figure 1a–c, 0 min).

**Figure 3:**
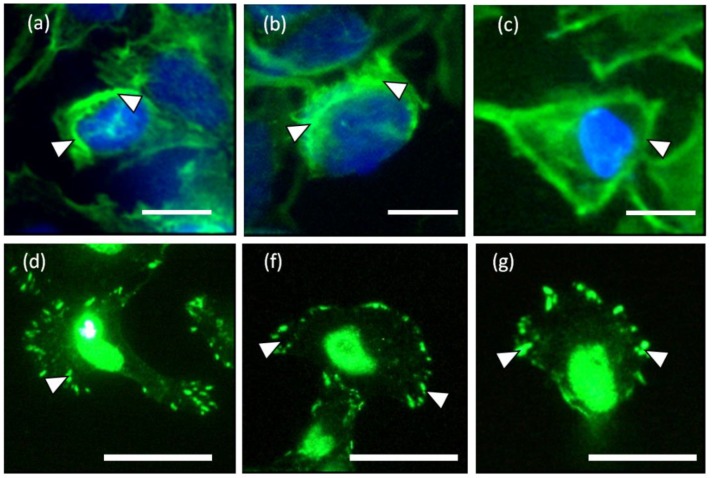
(**a**–**c**) Circumferential actin filaments and (**d**–**f**) vinculin accumulations located at the lamellipodia of the keratinocytes cultured on liquid crystal (LC) substrate. The white arrow heads indicate the short actin bundles and focal adhesions located at the circumference of the keratinocytes in (**a**–**c**) and (**d**–**g**), respectively. (Scale bar: 25 µm).

Leading edge of the cell was found developing into lamellipodia after 5 min of observation (Figure 1a–c, 5 min). The forces applied increased drastically from 25 to 110 nN (Figure 1b and Figure 2). At approximately 10 to 15 min of observation, the force at the leading edge dropped slightly to 90 nN and then to 80 nN (Figure 1a–c and Figure 2). The protrusion of the lamellipodia was coupled with formation of new deformation lines radiating up front. Actin and vinculin stained cells with similar morphology show that the protrusion could be associated with the organization of circumferential actin bundles and pointy focal adhesions distributed at the leading edge of the keratinocyte (Figure 3a,b). Previous literature also shows that migrating keratinocytes do express contractile circumferential actin [22,23,24]. The wrinkles observed is closely associated with the contraction of circumferential actin bundles leading to the generation of compressive forces exerted to the surface of the liquid crystals.

From 5 to 15 min of monitoring, the trailing end of the cell showed sign of retraction indicated by discharged of compressive forces and reduction of deformation lines (Figure 1). The decrease of forces from approximately 80 nN to 10 nN at the rear of the cell was in contrast to the leading edge (Figure 2). 

Overall, the leading edge, trailing end and flanks were playing roles in translocation of the single cell. Initially, the cell tried to break the symmetry of the cell morphology. As the trailing end of the cell retracted, the traction forces shifted from the trailing end of the cell tangentially along the boundary of the cell towards the right and left margins (Figure 1b, 5 and 10 min). The shift of forces increased gradually from the rear end to the flanks. The transverse traction force exerted by the flanks seemed to be coordinating with the rear of the cell in propelling the rear forward (Figure 1a–c, 5 and 10 min). This involved the broadening of the trailing end (Figure 1a–c, 10 and 15 min) and breaking the symmetrical morphology to an asymmetrical morphology. Such a physical restructuring is the characteristic of cell in the transition from non-polarized phenotype into polarized phenotype [25]. Towards the end of the translocation, the traction forces were much reduced. The cell remained in quiescent state for the next 45 min of monitoring. Over the 15 min of monitoring, the cell migrated a distance of 8 µm at a speed of approximately 0.5 µm/min. This speed is comparable to 0.6 µm/min as reported in [23] for keratinocytes.

The LC deformation line at the periphery of the cell and fluorescence staining of the actin distribution indicate that the contractile machineries for cell translocation are located at the periphery of the cell. However, cell traction forces were not detected at the central region of the cell body. The location of the contractile counterparts of keratinocytes is supported by both the actin and vinculin stainings as shown in Figure 3. Hence, the central region is hypothesized to be weakly adhered to the liquid crystal substrate enabling the transportation of the passive body load.

For cell to transform from a roundish morphology into migratory phenotype, cell locomotion is initiated at the trailing edge of a cell as indicated in the result of this work. LCTFT provides a means to elucidate this comprehensive mechanism and beyond. The cell locomotion process was suggested to be associated with perinuclear contractility leading to an increase in the F-actin flow at trailing edge towards the cell movement direction [26]. While these striated bundles of actin retract at the cell rear, they are probably accompanied by an expansion of actin bundles at the leading edge. For cell to form lamella, the circumferential actin bundles need to be disassembled and combined with the extended stress fibers radiated from the center of the cell body [24,27]. This is similar to the projection of lamella found for keratinocytes observed in Figure 1.

**Figure 4:**
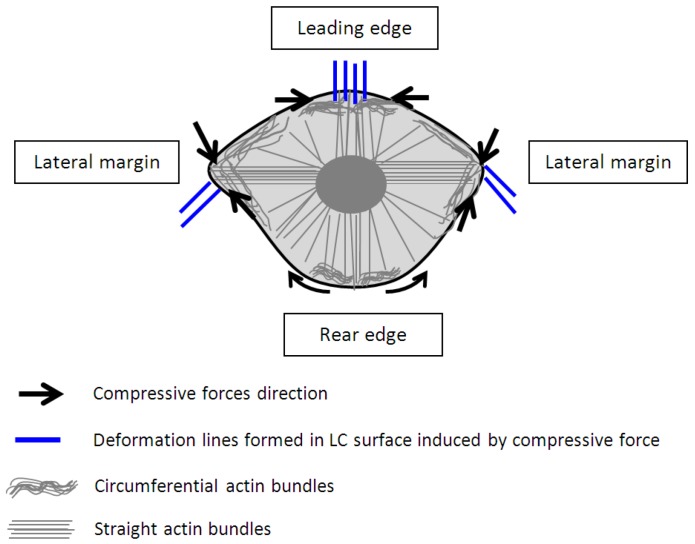
A model proposed for the contraction of circumferential and straight actin bundles for translocation of a keratinocyte associating with the deformation lines form in the liquid crystals.

Based on our observation on the translocation of a keratinocytes over a soft surface and the observation of time resolved transient cell forces (Figure 2) in addition to the established study on the structures and organization of actin filaments [24,27], we proposed a new model as shown in Figure 4 to describe the dynamic restructuring of actin cytoskeleton in association with the migration of a keratinocyte. The new observation found in this study is the contractions of the circumferential actin bundles along the lateral margins of the flanks generate strong forces pulling the rear of the cell body forward. While the straight actin bundles may function to protrude the cell body to anchor to new attachment area. The different organizations of the actin cytoskeleton function to regulate various cell contractile functions (breaking the cell symmetry, contraction, dynamic protrusion and retraction) and may enable the cell to exert variable forces on the LC surfaces in isolated regions via the focal adhesions. Such detailed observations were enabled using the liquid crystal based CTF measurement system.

**Figure 5:**
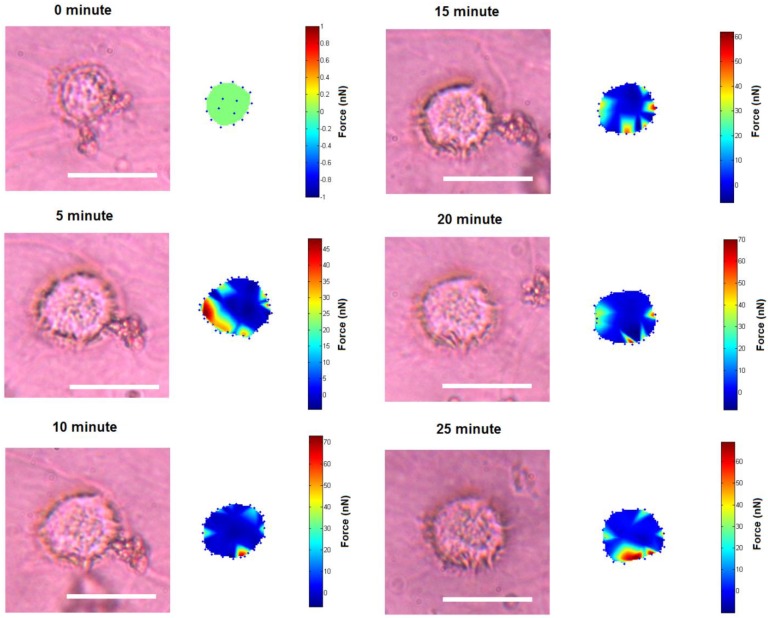
Small traction force changes of quiescent cell on the liquid crystal substrate (Scale bar: 20 µm).

Clearly, there are two types of actin organization in the keratinocytes which is the circumferential actin filaments and actin bundles within the cell body. The coordination between the two types of stress fibers in exerting forces to the surface of the liquid crystals could be associated with the deformation lines projected obliquely and parallel to the direction of migration. The oblique and parallel deformation lines were found at the lateral margins and leading edge of the cell, respectively. As illustrated in Figure 4, the disengaged circumferential filaments interrupted by the radiating actin bundles may contract and induce compressive forces that form the LC deformations lines located perpendicular to the protruded lamellipodia at the leading edge. Likewise, the short actin fibers located at the lateral margins contracted and induced perpendicular deformation lines on the LC surface. The contractile activities of the lateral margins halted when the retraction of the rear end completed (Figure 1a, 15 min). The deformation lines found at the leading edge continued protruding upon the finishing of the migration. However, not every keratinocytes behave similar and adopting the same motility pattern. Figure 5 is another example of traction forces exerted by a sedentary keratinocyte and measured using the LC based cell traction force transducer and microscopy system. The highly sensitive system detected small changes of forces (0–70 nN) at the periphery of the cell supporting the contractile activity of circumferential actin filament.

Apart from protrusion of actin fibers, new focal contacts need to be formed at the leading edge for mobilizing a cell. The adhesion of a cell via the integrin receptors to a surface is preceded by deposition of new ECM proteins [28,29]. These are the obligatory events required before the mechanical energy generated from the contractile and relaxation activity of the actin cytoskeleton can be delivered to the extracellular matrix or substrate. The development of lamellipodia, tension transmission of the cytoskeleton and re-adhesion of lamellipodia on the liquid crystal substrate are mediated by focal contacts and contractions of the myosin-II molecules [29,30,31].

For the retraction of the cell trailing end, the adhesion to the extracellular matrix must be detached by deployment of signaling to the integrin receptors and de-polymerization of the actin myosin molecules [32]. In this study, the withdrawn lamellipodia involved with more complex mechanism in which the actin filaments were de-bundled and possibly shortening of the dorsal filaments as reported in [33]. This would be followed by disengagement of focal contacts and detachment of lamellipodia from the extracellular matrix or substrate [33,34].

A study on time-dependent traction force microscopy of cancer cells involving with fluorescence markers was recently reported in [6] but the time dependent forces reported was average forces of cells over time, lacking the information associating with localized traction force map of a single cell with respect to time. As compared to the migration forces (0–23 nN) of cancer cells on polyacrylamide gel at a stiffness of 10 kPa [6], the traction forces (0–160 nN) of the keratinocyte reported in this work is higher which is probably due to the lower compliance of liquid crystal used (87.1 ± 17.2 kPa). This could be possible because cells tend to expressed higher traction stresses in accommodating to stiffer substrate and *vice versa* [35]. However, the liquid crystals used in this work having a stiffness approximates the stiffness of the human skin and epidermis layer [36,37] may be more suitable in studying the traction forces of human keratinocytes.

## 4. Conclusions

In conclusion, the findings of this study indicate that LCTFT enables the time resolved tracking of traction forces associated with the migration of a keratinocyte. This work has also revealed more insights on the specific roles played by different facets of the keratinocyte actin cytoskeleton especially the circumferential actin filaments, during the translocation of a keratinocyte from the quiescent state to a migrating state on a liquid crystal surface. Cell migration is very much modulated by up-regulation of a range of cytokines. Determining the exact role of the cytokines in the mechanics of cell migration is difficult. However, it is possible that the combination of the LCTFT and CTFM software system along with the use of a fast image capture system may enable the real-time imaging and analysis of traction forces during cell migration. This could allow the pharmacological role of cytokines to be examined in more detail, which in turn could potentially have implication in the assaying of pharmaceuticals products targeted at enhancing wound repair.

## Supplementary Files

Supplementary File 1
